# Atopic Disease Development in Offspring Conceived via Assisted Reproductive Technology

**DOI:** 10.1001/jamanetworkopen.2025.51690

**Published:** 2025-12-30

**Authors:** Yao-Chi Hsieh, Ching-Heng Lin, Ming-Chih Lin, Yi-Hsuan Lin

**Affiliations:** 1 Department of Pediatrics, Kuang Tien General Hospital, Taichung, Taiwan; 2 Department of Medical Research, Taichung Veterans General Hospital, Taichung, Taiwan; 3 Institute of Public Health and Community Medicine Research Center, National Yang Ming Chiao Tung University, Taipei, Taiwan; 4 Department of Epidemiology and Public Health, UCL, London, United Kingdom; 5 Department of Post-Baccalaureate Medicine, College of Medicine, National Chung Hsing University, Taichung, Taiwan; 6 Division of Neonatology, Children’s Medical Center, Taichung Veterans General Hospital, Taichung, Taiwan; 7 School of Medicine, National Yang Ming Chiao Tung University, Taipei, Taiwan; 8 School of Medicine, Chung Shan Medical University, Taichung, Taiwan

## Abstract

**Importance:**

As assisted reproductive technology (ART) becomes increasingly prevalent, concerns regarding the potential short-term and long-term health outcomes of children conceived through these methods have emerged.

**Objective:**

To investigate whether conception via ART is associated with a risk of atopic disease development in offspring.

**Design, Setting, and Participants:**

This retrospective, population-based cohort study was conducted using data obtained from Taiwan’s National Health Insurance Research Database, Assisted Reproduction Database, and Maternal and Child Health Database. Children conceived via ART and born between January 1, 2004, and December 31, 2014, were identified as the exposure group. A control group of children conceived naturally was selected using 1:4 matching based on maternal age, neonatal sex, and birth month. Data analysis was performed from December 1, 2023, to November 1, 2025.

**Exposure:**

Conception via ART or naturally.

**Main Outcomes and Measures:**

The primary outcome was development of atopic disease. Both groups were followed up until December 31, 2020. Cox proportional hazards regression models were used to adjust potential confounders.

**Results:**

A total of 69 785 children (13 957 in the ART group and 55 828 in the control group) were included in this study, with follow-up beginning at birth. The study population was 47.5% female and 52.5% male. For the ART and control groups, the mean (SD) follow-up duration was 7.99 (4.22) and 8.41 (4.18) years for asthma, 5.79 (4.12) and 6.34 (4.28) years for allergic rhinitis, and 7.34 (5.13) and 7.62 (5.14) years for atopic dermatitis, respectively. After adjusting for potential confounders, the ART group exhibited a higher likelihood of developing asthma (adjusted hazard ratio [AHR], 1.13 [95% CI, 1.09-1.18]), allergic rhinitis (AHR, 1.15 [95% CI, 1.12-1.18]), or atopic dermatitis (AHR, 1.08 [95% CI, 1.05-1.12]) (all *P* < .001) compared with the control group. Subgroup analysis revealed that children conceived with fresh embryos faced a greater risk of developing allergic rhinitis compared with those conceived with frozen embryos (AHR, 1.12 [95% CI, 1.06-1.19]; *P* < .001).

**Conclusions and Relevance:**

The findings of this cohort study suggest that children conceived via ART had a higher risk of developing asthma, allergic rhinitis, or atopic dermatitis. These findings underscore the importance of long-term follow-up for offspring conceived via ART and further investigation into the underlying biological mechanisms by which ART may contribute to atopic disease development.

## Key Points

**Question:**

Is conception via assisted reproductive technology (ART) associated with an increased risk of atopic disease development in offspring?

**Findings:**

In this cohort study of 69 785 children born in Taiwan between 2004 and 2014, those conceived through ART had a significantly higher risk of developing asthma, allergic rhinitis, or atopic dermatitis compared with those conceived naturally.

**Meaning:**

These findings highlight the need for long-term surveillance of children conceived via ART as well as a need for further research into the underlying mechanisms by which ART may contribute to atopic disease development.

## Teaser

This cohort study examines whether conception via assisted reproductive technology is associated with an increased risk of atopic disease development in offspring.

## Introduction

As assisted reproductive technology (ART) continues to advance, there has been a gradual increase in the numbers of children born using these methods. It is estimated that ART currently represents 1% to 4% of all births, especially in high-income societies.^1,2^ Moreover, techniques within ART, such as fresh and frozen embryo transfer, are also experiencing rapid advancements.^1,3^ Consequently, concerns regarding the potential short-term and long-term health outcomes of children conceived through these methods have emerged.^2,4,5^

Asthma, recognized as the most prevalent chronic disease in children, imposes a substantial burden on caregiving responsibilities, reduces school attendance, and diminishes overall quality of life.^6,7^ Atopic diseases are believed to be influenced by genetic factors and environmental triggers.^8,9^ According to the developmental origins of health and disease (DOHAD) theory, various factors in fetal stages could contribute to programmed changes in the structure and function of organs and tissues. These changes can affect the onset and progression of chronic diseases in adulthood, such as diabetes, metabolic syndrome, and cardiovascular disease.^10^ However, whether the use of ART affects children’s long-term risk of developing atopic diseases remains controversial. Some studies have suggested that ART is associated with a higher risk of developing asthma,^11,12,13,14,15^ whereas others have found no association between conception via ART and development of asthma.^16,17,18,19^ Moreover, it has been reported that individuals conceived through ART may experience milder asthma symptoms until adulthood.^20^ Although a 2024 study^19^ reported no association between ART use and development of asthma, its interpretation is limited by the small sample size and single-center design. The present study aimed to address this question using a large-scale, population-based approach. This study investigated, from a population-based perspective, whether children conceived via ART had a higher likelihood of developing atopic diseases compared with children conceived naturally.

## Methods

This cohort study was approved by the Taichung Veterans General Hospital Institutional Review Board, which waived the requirement for informed consent due to the use of deidentified data. The study followed the Strengthening the Reporting of Observational Studies in Epidemiology (STROBE) reporting guideline.

### Data Sources

The main data source was the National Health Insurance Research Database (NHIRD), which was launched in 1995 and currently covers nearly the entire population of Taiwan (99.99% of 23.5 million).^21,22^ To match mothers’ data and children’s data, we also linked the NHIRD with the Maternal and Child Health Database,^23^ which is maintained by Taiwan’s Health Promotion Administration. The ART data were collected from the Assisted Reproduction Database of Taiwan (ARD). Starting in 2007, the Ministry of Health and Welfare mandated that all clinics and hospitals involved in ART must contribute data to these databases. According to the ARD, cases are included in the database if they involve procedures such as in vitro fertilization (IVF) and embryo transfer, intracytoplasmic sperm injection (ICSI), gamete intrafallopian transfer, and zygote intrafallopian transfer and tubal embryo transfer. In contrast, procedures involving intrauterine insemination and ovulation induction alone are not included in the ARD.

### Study Population

**Figure 1.**
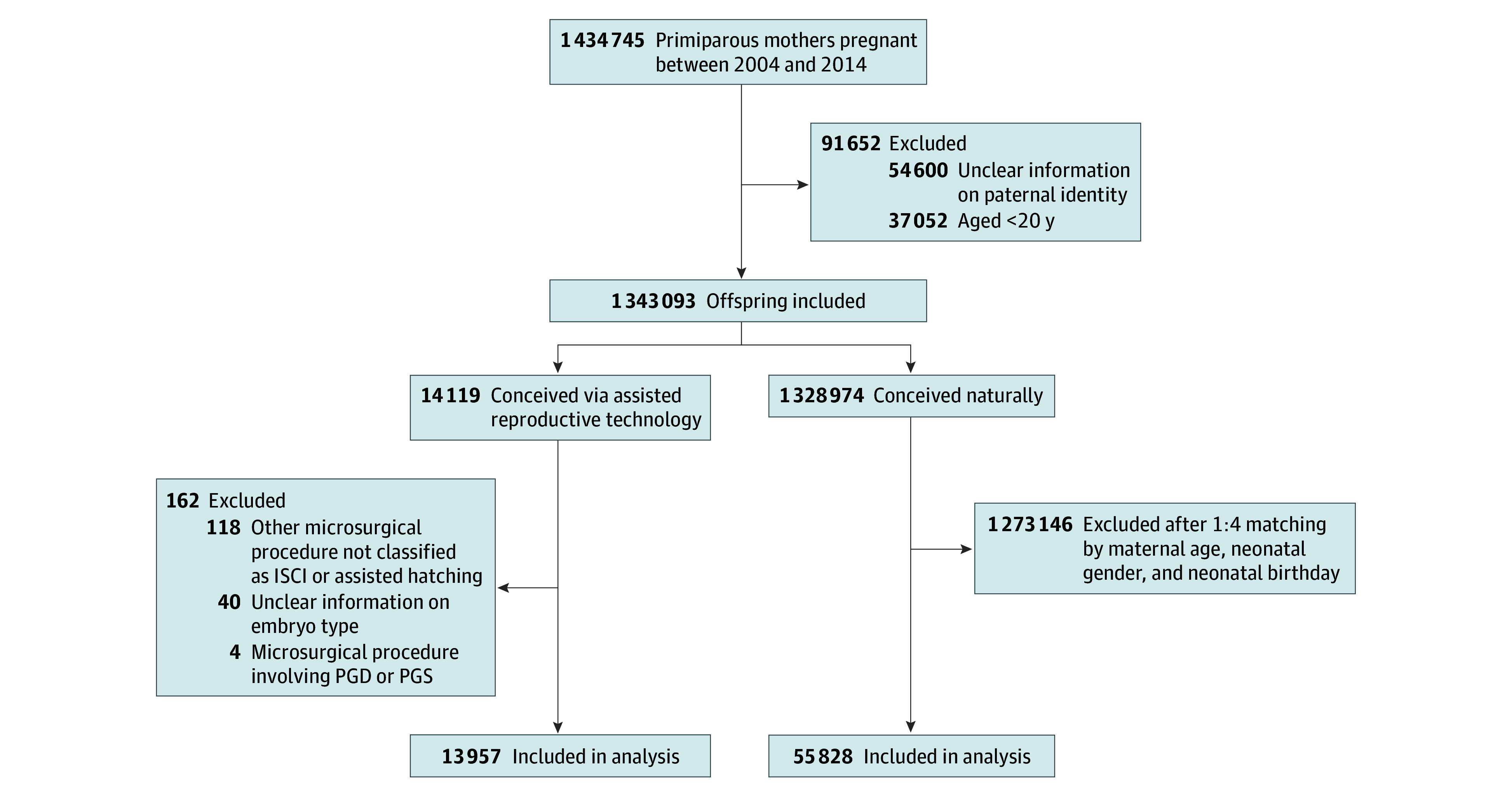
Study Flow Diagram ICSI indicates intracytoplasmic sperm injection; PGD, preimplantation genetic diagnosis; PGS, preimplantation genetic screening.

In this study, we included only children who were their mother’s first live-born child during the study period (January 1, 2004, to December 31, 2014); second or subsequent births were excluded. In cases of multiple births (eg, twins or higher-order multiples), only 1 child per mother was selected to ensure that each mother contributed only 1 child to the analysis. This approach was adopted to avoid intrafamily clustering, which could introduce bias. A total of 1 434 745 first-born offspring (1 per mother) were included during the study period (Figure 1). After excluding offspring whose mothers were aged younger than 20 years (n = 37 052) or who had indeterminate paternal identity (n = 54 600), the study cohort comprised 1 343 093 offspring. We further stratified them into a control (non-ART) group (n = 1 328 974) and an ART group (n = 14 119). Within the ART group, additional exclusions were applied as follows: cases involving preimplantation genetic diagnosis (PGD) or preimplantation genetic screening (PGS) (n = 4); other microsurgical procedures not classified as ICSI or assisted hatching (n = 118); and instances with incomplete or ambiguous information regarding embryo type (n = 40). During the study period, PGD and PGS were still relatively new technologies in Taiwan, and their clinical application was limited. As a result, only a small number of such cases were recorded in the national database. To avoid potential confusion, PGD and PGS cases were excluded from the analysis. These exclusions yielded a final ART group of 13 957 individuals. The control group was selected from the natural conception cohort using a 1:4 ratio and matching by maternal age, neonatal sex, and birth month. Mothers in the control group had no prior history of undergoing ART. This matching process resulted in the inclusion of 55 828 children in the control group.

### Exposures and Outcomes

Diagnostic data for children were collected using both *International Classification of Diseases, Ninth Revision, Clinical Modification* (*ICD-9-CM*) codes and *International Classification of Diseases, Tenth Revision, Clinical Modification* (*ICD-10-CM*) codes (Taiwan’s National Health Insurance system used *ICD-9-CM* codes until 2016 and transitioned to *ICD-10-CM* codes starting in 2017). Diagnoses of asthma, atopic dermatitis, or allergic rhinitis were ascertained based on consistent primary diagnostic codes recorded during a single inpatient admission or across a minimum of 3 outpatient encounters. A diagnosis of atopic dermatitis, asthma, or allergic rhinitis was confirmed if the child had either (1) at least 1 inpatient admission with the corresponding *ICD-9-CM* or *ICD-10-CM* code or (2) 3 or more outpatient visits with the same diagnosis code. For diagnoses based on hospitalization, the diagnosis date was defined as the admission date of the first relevant hospital stay. For diagnoses based on outpatient visits, the diagnosis date was defined as the date of the first outpatient encounter with the relevant code. The 3 atopic conditions—asthma, atopic dermatitis, and allergic rhinitis—were analyzed and reported individually. As such, a child could be diagnosed with 1, 2, or all 3 conditions during the follow-up period. The presence of one condition (eg, allergic rhinitis) does not preclude the development of another (eg, asthma or atopic dermatitis). The following *ICD-9-CM* and *ICD-10-CM* diagnosis codes were used, respectively: 493 and J45 for asthma; 477 and J30 for allergic rhinitis; 691 and L20 for atopic dermatitis; 642.3 as well as O13 and O16 for gestational hypertension; 624.4, 624.5, and 624.6 as well as O11, O14, and O15 for preeclampsia or eclampsia; and 648.0 and 648.8 as well as O24 for gestational diabetes. The specific *ICD-9-CM* and *ICD-10-CM* diagnostic codes used in this analysis are detailed in eTable 1 in Supplement 1. Maternal and neonatal factors with potential confounding effects were collected, including neonatal sex, gestational age, birth weight, mode of delivery, maternal age, urbanization, family income, maternal and paternal histories of atopic diseases, and pregnancy-related complications. All children were longitudinally followed up until December 31, 2020. In the survival analysis, the time to event was defined as the interval from birth to the first diagnosis of atopic disease. Children were censored at death or at the end of the study period (December 31, 2020). Given that the NHIRD encompasses more than 99.9% of the Taiwanese population within a universal health care system, loss to follow-up was considered negligible.

### Covariates

All confounder data were obtained from the NHIRD. Family income was determined based on the insurance premium category assigned to the family at the time of the child’s birth, which serves as a standardized proxy for socioeconomic status within the NHIRD. Parental history of atopic diseases—including asthma, allergic rhinitis, and atopic dermatitis—was identified using diagnostic records from the NHIRD. A parental diagnosis was defined as present if there was at least 1 inpatient admission or if there were 3 or more outpatient visits with the corresponding *ICD-9-CM* or *ICD-10-CM* codes during the study period. Detailed information is available in eTable 2 in Supplement 1.

To clarify the assumed causal pathways between the exposure and outcomes, we constructed a directed acyclic graph (DAG) to illustrate our underlying assumptions. We identified parental history of atopic diseases (as a proxy for genetic predisposition), socioeconomic status, and urbanization level as potential confounders. These variables were included in the Cox proportional hazards regression models and are represented as gray-shaded rectangular nodes in the DAG (eFigure 1 in Supplement 1). To estimate the direct effect of ART on the risk of atopic disease development, independent of established causal pathways such as prematurity, we also adjusted for several mediators. These included maternal pregnancy-related morbidities, prematurity or low birth weight, mode of delivery, multiple birth, and child’s sex. These mediators are depicted as gray-shaded elliptical nodes in eFigure 1 in Supplement 1.

### Statistical Analysis

Demographic characteristics are summarized as frequencies and percentages. Categorical variables were compared using a χ^2^ test. Cumulative incidences of atopic diseases, including asthma, allergic rhinitis, and atopic dermatitis, were estimated using Kaplan-Meier survival analysis. To adjust for potential confounding factors, multivariable Cox proportional hazards regression models were used. Subgroup analyses were performed to evaluate whether ICSI use and embryo type (frozen or fresh) were associated with the incidence of atopic diseases. We also conducted interaction tests for subgroup analyses using Cox proportional hazards regression models. Two-sided *P* < .05 was considered statistically significant. Statistical analyses were performed using SAS, version 9.4 (SAS Institute). Data analysis was performed from December 1, 2023, to November 1, 2025.

## Results

A total of 69 785 children (13 957 in the ART group and 55 828 in the control group) were included in this study, with follow-up beginning at birth. The study population was 47.5% female and 52.5% male. For the ART and control groups, the mean (SD) follow-up duration was 7.99 (4.22) and 8.41 (4.18) years for asthma, 5.79 (4.12) and 6.34 (4.28) years for allergic rhinitis, and 7.34 (5.13) and 7.62 (5.14) years for atopic dermatitis, respectively. When comparing the baseline characteristics between groups, the ART group exhibited a markedly higher prevalence of parental history of allergic rhinitis in both paternal (26.4% vs 22.3%; *P* < .001) and maternal (32.2% vs 29.1%; *P* < .001) lineages than the control group. Furthermore, maternal complications were more prevalent within the ART group, including gestational diabetes (2.9% vs 1.7%; *P* < .001) and preeclampsia or eclampsia (1.0% vs 0.4%; *P* < .001). The ART group was also characterized by a significantly higher rate of cesarean delivery (65.6% vs 42.0%; *P* < .001). Regarding neonatal outcomes, the incidence of multiple births was significantly elevated in the ART group (30.5% vs 2.0%; *P* < .001). Additionally, the ART group exhibited a higher proportion of low birth weight (<2500 g) (27.4% vs 7.9%; *P* < .001) and a markedly higher risk of preterm birth (<37 weeks of gestation) (29.0% vs 9.1%; *P* < .001). These baseline characteristics are summarized in the Table.

**Table.** Cohort Characteristics^a^

Characteristic	Study group		*P* value
	Natural conception (control) (n = 55 828)	Assisted reproductive technology (n = 13 957)	
Maternal age, y			
20-24	364 (0.7)	91 (0.7)	>.99
25-29	5464 (9.8)	1366 (9.8)	
30-34	23 612 (42.3)	5903 (42.3)	
≥35	26 388 (47.3)	6597 (47.3)	
Family income, $			
≤18 780	11 870 (21.3)	2024 (14.5)	<.001
18 781-27 600	17 956 (32.2)	4285 (30.7)	
27 601-42 000	13 300 (23.8)	3106 (22.3)	
>42 000	12 702 (22.8)	4542 (32.5)	
Urbanization			
Urban	37 107 (66.5)	9428 (67.6)	.05
Suburban	6096 (10.9)	1490 (10.7)	
Rural	12 625 (22.6)	3039 (21.8)	
History of paternal diseases			
Asthma	2026 (3.6)	508 (3.6)	.95
Allergic rhinitis	12 461 (22.3)	3691 (26.4)	<.001
Atopic dermatitis	896 (1.6)	274 (2.0)	.003
History of maternal diseases			
Asthma	2414 (4.3)	582 (4.2)	.42
Allergic rhinitis	16 256 (29.1)	4497 (32.2)	<.001
Atopic dermatitis	2221 (4.0)	554 (4.0)	.96
Pregnancy-related complications			
Gestational hypertension	67 (0.1)	25 (0.2)	.09
Gestational diabetes	976 (1.7)	402 (2.9)	<.001
Preeclampsia or eclampsia	204 (0.4)	139 (1.0)	<.001
Mode of delivery			
Vaginal	32 407 (58.0)	4806 (34.4)	<.001
Cesarean	23 421 (42.0)	9151 (65.6)	
Neonatal sex			
Female	26 536 (47.5)	6634 (47.5)	>.99
Male	29 292 (52.5)	7323 (52.5)	
No. of children			
Singleton	54 693 (98.0)	9705 (69.5)	<.001
Multiple	1135 (2.0)	4252 (30.5)	
Birth weight, g			
≥3500	9394 (16.8)	1345 (9.6)	<.001
3000-3499	24 846 (44.5)	4251 (30.5)	
2500-2999	17 157 (30.7)	4533 (32.5)	
<2500	4431 (7.9)	3828 (27.4)	
Gestational age, wk			
≥37	50 750 (90.9)	9905 (71.0)	<.001
32-36 + 6 d	4102 (7.3)	3109 (22.3)	
26-31 + 6 d	821 (1.5)	803 (5.8)	
<26	155 (0.3)	140 (1.0)	

^a^ Values are presented as No. (%) of offspring.

### Association Between ART and Atopic Disease Development

**Figure 2.**
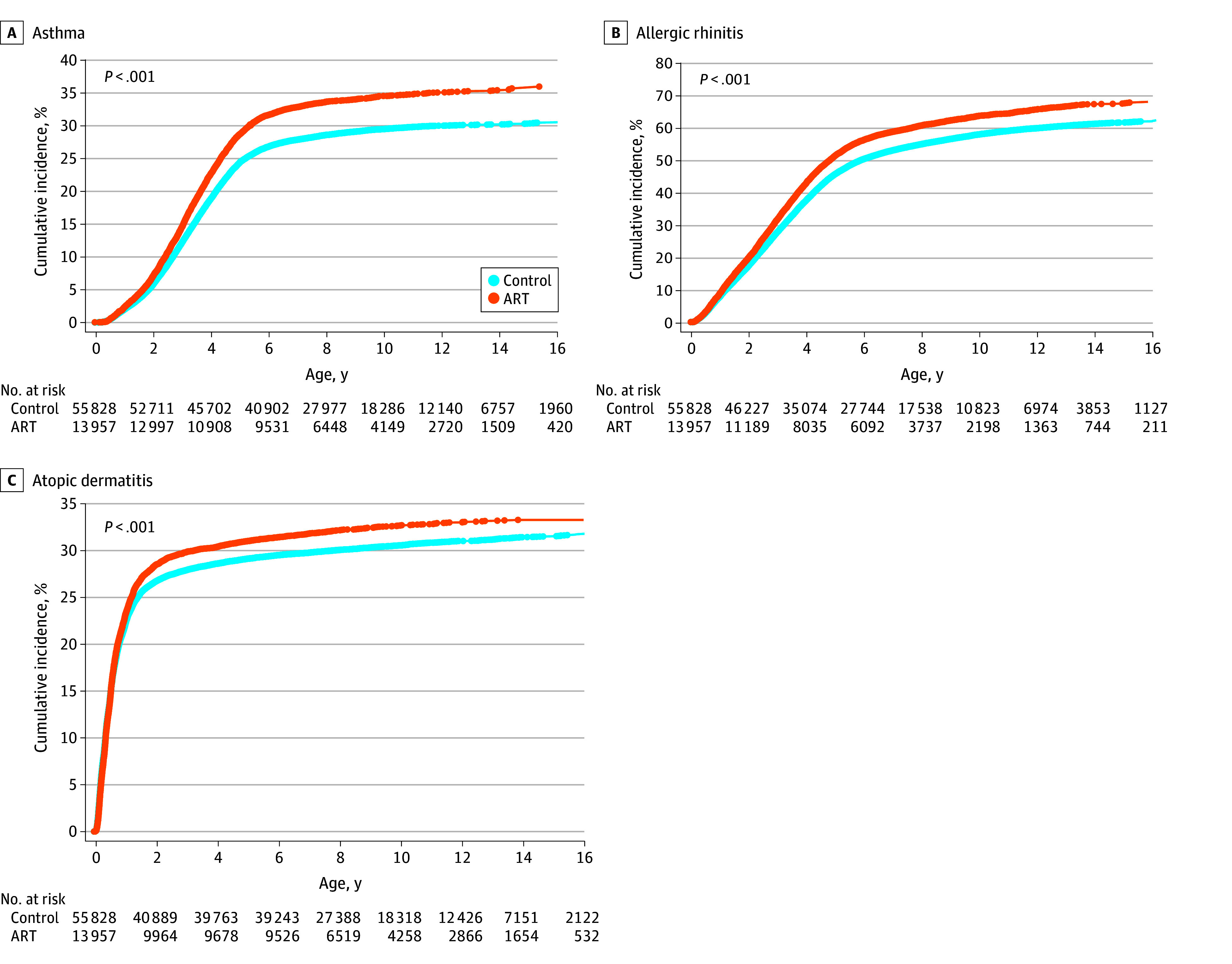
Cumulative Incidence of Atopic Diseases in Children Conceived Naturally or via Artificial Reproductive Technology (ART)

**Figure 3.**
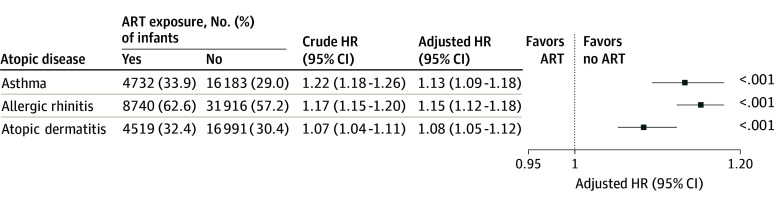
Risk of Atopic Disease Development Among Offspring Conceived With Artificial Reproductive Technology (ART) The model was adjusted for family income, urbanization, history of paternal atopic disease, history of maternal atopic disease, pregnancy-related complication, mode of delivery, neonatal sex, multiple births, birth weight, and gestational age. HR indicates hazard ratio.

Children conceived via ART exhibited significantly higher cumulative incidences of atopic diseases compared with those conceived naturally. Specifically, children conceived via ART demonstrated a higher likelihood of developing asthma (crude hazard ratio [CHR], 1.22 [95% CI, 1.18-1.26]; *P* < .001), allergic rhinitis (CHR, 1.17 [95% CI, 1.15-1.20]; *P* < .001), or atopic dermatitis (CHR, 1.07 [95% CI, 1.04-1.11]; *P* < .001) (Figure 2). Multiple Cox proportional hazards regression models were used to adjust for potential confounding variables, including family income, urbanization level, parental history of atopic diseases, pregnancy-related complications, mode of delivery, neonatal sex, multiplicity of birth, birth weight, and gestational age. After adjustment, ART conception was associated with an elevated adjusted hazard ratio (AHR) for the development of asthma (AHR, 1.13 [95% CI, 1.09-1.18]; *P* < .001), allergic rhinitis (AHR, 1.15 [95% CI, 1.12-1.18]; *P* < .001), or atopic dermatitis (AHR, 1.08 [95% CI, 1.05-1.12]; *P* < .001) (Figure 3). These findings suggest an association between ART conception and a higher risk of atopic disease development, independent of various demographic, obstetric, and neonatal confounders.

### Association Between Microsurgical Procedures and Embryo Type

To investigate the potential association between microsurgical procedures, such as ICSI and embryo type (fresh vs frozen), and incidence of atopic diseases in children conceived via ART, multiple Cox proportional hazards regression models were used. These models were adjusted for potential confounders, including family income, urbanization level, parental history of atopic diseases, pregnancy-related complications, mode of delivery, neonatal sex, multiplicity of birth, birth weight, and gestational age.

The use of ICSI was not associated with a notable difference in the risk of developing asthma (AHR, 1.04 [95% CI, 0.98-1.10]; *P* = .18), allergic rhinitis (AHR, 0.99 [95% CI, 0.95-1.03]; *P* = .68), or atopic dermatitis (AHR, 1.04 [95% CI, 0.98-1.11]; *P* = .17). These findings suggest that ICSI, as a microsurgical technique, does not confer a higher risk of atopic disease development among children conceived via ART (eFigure 2 in Supplement 1).

In comparisons between fresh and frozen embryo transfers, children conceived using fresh embryos exhibited a significantly higher risk of developing allergic rhinitis (AHR, 1.12 [95% CI, 1.06-1.19]; *P* < .001). However, no statistically significant differences between groups were observed for risk of developing asthma (AHR, 0.96 [95% CI, 0.89-1.05]; *P* = .37) or atopic dermatitis (AHR, 1.01 [95% CI, 0.93-1.10]; *P* = .79) (eFigure 3 in Supplement 1). No significant interaction between ICSI and embryo type was observed for asthma, allergic rhinitis, or atopic dermatitis (eTable 3 in Supplement 1). These findings suggest that although ICSI use was not associated with the risk of atopic disease development, fresh embryo transfer may be associated with an elevated risk of allergic rhinitis.

## Discussion

In this large-scale nationwide cohort study, we analyzed data from the NHIRD to assess whether conception via ART is associated with the risk of atopic disease development in offspring. The results suggest that children conceived using ART are more likely to develop atopic diseases, including asthma, allergic rhinitis, and atopic dermatitis, compared with those conceived naturally.

In the literature, it remains controversial as to whether ART is associated with atopic disease development among offspring. Some studies have suggested that children conceived through ART are more likely to develop asthma.^11,12,13,14,15^ However, other reports have found no substantial difference in the incidence of atopic diseases between children conceived via ART and children conceived naturally.^16,17,18,19^ Additionally, some experts have proposed that children conceived via ART may experience better asthma outcomes in adulthood.^20^

The DOHAD theory suggests that various factors influencing early human development, including the fetal stage, can contribute to permanent changes in the structure and function of organs and tissues, which can subsequently affect the onset and progression of chronic diseases.^10^ This theory may help explain our study findings suggesting that the use of ART technology may contribute to the development of atopic disease in children. Moreover, the potential effects of ART technology on the immune system have been the subject of research efforts. Studies showed that mice conceived through ART exhibited a weaker response to the Bacillus Calmette-Guérin vaccine and increased promotion of Th2 immune responses.^24,25^ Moreover, children conceived using ART had a higher risk of developing cancer, suggesting a higher immune tolerance to tumor antigens.^26^ However, the mechanisms by which ART may contribute to atopic disease development remain unclear. Current research found that children conceived using ART had higher levels of interferon-γ and interleukin-4 in their serum; however, the ratio of interferon-γ to interleukin-4 was lower in children conceived via ART compared with those conceived naturally.^27^

We conducted a further subanalysis to examine outcomes associated with conception via ART. First, regarding microsurgical procedures, we found that the use of ICSI was not associated with the risk of developing asthma, allergic rhinitis, or atopic dermatitis in children. This finding is consistent with a previous study comparing ICSI and IVF, which reported no difference in the incidence of asthma and eczema among children followed up to age 5 to 8 years.^28^ Second, we compared the use of frozen embryos vs fresh embryos in ART. There was no significant difference in the incidence of asthma and atopic dermatitis between these 2 groups. All subgroup analyses were restricted to the population conceived via ART. These analyses aimed to explore whether specific ART-related procedures, such as ICSI use and embryo transfer type (fresh vs frozen), were associated with differential risks of atopic diseases among children conceived via ART. In this analysis, we observed a higher likelihood of allergic rhinitis among children conceived through fresh embryo transfer. This finding contrasts with previous studies,^29,30^ which have generally reported no substantial differences in respiratory or immune-related outcomes between offspring conceived via fresh and frozen embryo transfers. Currently, no established biological mechanism or supporting literature is available to explain this association. As such, this observation may have occurred by chance. Further investigation is warranted to determine whether this finding reflects a true effect or is due to random variation.

Previous studies reported that mothers who undergo ART have a higher risk of experiencing pregnancy-related complications, such as gestational diabetes and higher rates of cesarean delivery.^31,32^ Furthermore, children conceived via ART are more likely to be born preterm, to have lower birth weight, and to be part of a multiple birth compared with those conceived naturally.^32,33,34,35,36^ These findings are consistent with the results of our study. In this study, the ART group was characterized by a higher average family income. A prior Japanese study identified low family income as a risk factor for the development of asthma and eczema.^37^ Although adjustments for potential confounders were performed using multivariable Cox proportional hazards regression models in this study, the possibility of residual confounding due to unmeasured variables cannot be entirely excluded. Through this framework, we identified a notably higher proportion of multiple births in the ART group compared with the control group (30.5% vs 2.0%; *P* < .001) (Table). Given that multiple births are known to be associated with a higher likelihood of preterm birth and lower birth weight (both of which are well-established risk factors for asthma and other atopic diseases), we considered whether this imbalance might bias our findings.

The high proportion of multiple births in the ART group vs the control group (30.5% vs 2.0%) may have contributed to differences in baseline characteristics, and adjusting for gestational age and birth weight could introduce collider bias. To address this issue, we conducted sensitivity analyses stratified by plurality, gestational age, and birth weight. The associations between ART and the cumulative risk of developing asthma, allergic rhinitis, or atopic dermatitis remained statistically significant after excluding multiple births. However, the results within the multiple birth stratum were not statistically significant, which may be attributable to the limited number of cases in this subgroup. Additionally, when gestational age and birth weight were adjusted separately, the associations between ART and atopic diseases remained consistent (eTable 4 in Supplement 1). Information on triplet and quadruplet births is presented in eTable 5 in Supplement 1; however, due to the very limited number of such cases in the database, further analysis could not be performed.

In Taiwan, health care access is highly convenient. There is no mandatory referral system, and patients can visit emergency departments or consult specialists directly without first seeing a primary care physician. In our study, both emergency department visits and specialist consultations were classified as outpatient encounters. To enhance diagnostic validity and reduce the risk of misclassification, we adopted a threshold of 3 outpatient visits. This approach is consistent with previous studies using the NHIRD.^38^ Because ambulatory care is easily accessible in Taiwan, most diagnoses of asthma, atopic dermatitis, and allergic rhinitis are made in outpatient settings.

### Limitations

This study has certain limitations. First, the NHIRD lacks laboratory findings and genetic information, which could have provided deeper insights into underlying mechanisms. Second, disease identification was based on physician-coded diagnoses, which may be prone to inaccuracies or inconsistencies, potentially leading to misclassification bias. Third, crucial environmental and lifestyle variables, such as dietary habits, physical activity levels, hygiene practices, and exposure to air pollution during pregnancy, were not captured in the dataset. Fourth, regarding the recurrent nature of atopic diseases such as asthma, this study focused on the diagnosis of asthma, atopic dermatitis, and allergic rhinitis by comparing their cumulative incidence. Although asthma is indeed a recurrent condition, the NHIRD claims data include visits for prescription refills and routine follow-up visits, which may not accurately reflect true recurrence or disease severity. Recurrent event analysis could provide additional insights and offers a potential direction for future research. Fifth, this analysis did not include medication data. Incorporating such data could provide further insight into this issue and may serve as a potential direction for future research. Finally, we attempted to address confounding factors by adjusting for relevant clinical and demographic variables, including pregnancy-related complications, maternal health conditions, and perinatal outcomes; however, the possibility of residual confounding due to unmeasured or inadequately recorded factors cannot be entirely ruled out.

## Conclusions

In this cohort study of 69 785 children conceived naturally or via ART, the ART group had a higher risk of developing asthma, allergic rhinitis, or atopic dermatitis. These findings underscore the importance of long-term follow-up for offspring conceived via ART and further investigation into the underlying biological mechanisms.

## Notes

Supplement 1. **eTable 1.** Specific *ICD-9-CM* and *ICD-10-CM* Diagnostic Codes Employed in This Analysis **eTable 2.** Definitions and Measurement of Covariates **eTable 3.** Interaction Tests for Subgroup Analyses of ICSI Use, Embryo Type, and Their Interaction With Risk of Atopic Disease Development Using Cox Proportional Hazards Regression Models **eTable 4.** Sensitivity Analyses of the Association Between Artificial Reproductive Technology and Atopic Diseases Stratified by Singleton and Multiple Births, With Alternative Adjustments for Gestational Age and Birth Weight **eTable 5.** Distribution of Triplet and Quadruplet Births in the Assisted Reproductive Technology (ART) and Control Groups **eFigure 1.** A Comprehensive Overview of the Complex Causal Relationships Between Assisted Reproductive Technology (ART) Exposure and the Development of Atopic Diseases **eFigure 2.** Hazard Ratio of Developing Atopic Diseases in Relation to Intracytoplasmic Sperm Injection (ICSI), Adjusted for Cofactors by Cox Proportional Hazards Regression Models (Analysis Limited to ART-Conceived Children) **eFigure 3.** Hazard Ratio of Developing Atopic Diseases in Relation to Fresh or Frozen Embryo, Adjusted for Cofactors by Cox Proportional Hazards Regression Models (Analysis Limited to ART-Conceived Children)

Supplement 2.
Data Sharing Statement
