# Supplementary material for: Atopic Disease Development in Offspring Conceived via Assisted Reproductive Technology
Source: JAMA Netw Open. 2025 Dec 30;8(12):e2551690. doi: 10.1001/jamanetworkopen.2025.51690 (PMC12754677; doi:10.1001/jamanetworkopen.2025.51690)
Supplement: Supplement 1. — eTable 1. Specific ICD-9-CM and ICD-10-CM Diagnostic Codes Employed in This Analysis eTable 2. Definitions and Measurement of Covariates eTable 3. Interaction Tests for Subgroup Analyses of ICSI Use, Embryo Type, and Their Interaction With Risk of Atopic Disease Development Using Cox Proportional Hazards Regression Models eTable 4. Sensitivity Analyses of the Association Between Artificial Reproductive Technology and Atopic Diseases Stratified by Singleton and Multiple Births, With Alternative Adjustments for Gestational Age and Birth Weight eTable 5. Distribution of Triplet and Quadruplet Births in the Assisted Reproductive Technology (ART) and Control Groups eFigure 1. A Comprehensive Overview of the Complex Causal Relationships Between Assisted Reproductive Technology (ART) Exposure and the Development of Atopic Diseases eFigure 2. Hazard Ratio of Developing Atopic Diseases in Relation to Intracytoplasmic Sperm Injection (ICSI), Adjusted for Cofactors by Cox Proportional Hazards Regression Models (Analysis Limited to ART-Conceived Children) eFigure 3. Hazard Ratio of Developing Atopic Diseases in Relation to Fresh or Frozen Embryo, Adjusted for Cofactors by Cox Proportional Hazards Regression Models (Analysis Limited to ART-Conceived Children) [file jamanetwopen-e2551690-s001.pdf]

Supplemental Online Content

Hsieh YC, Lin CH, Lin MC, Lin YH. Atopic disease development in offspring conceived via assisted reproductive technology. *JAMA Netw Open*. 2025;8(12):e2551690. doi:10.1001/jamanetworkopen.2025.51690

eTable 1. Specific *ICD-9-CM* and *ICD-10-CM* Diagnostic Codes Employed in This Analysis

eTable 2. Definitions and Measurement of Covariates

eTable 3. Interaction Tests for Subgroup Analyses of ICSI Use, Embryo Type, and Their Interaction With the Risk of Atopic Disease Development Using Cox Proportional Hazards Regression Models

eTable 4. Sensitivity Analyses of the Association Between Artificial Reproductive Technology and Atopic Diseases Stratified by Singleton and Multiple Births, With Alternative Adjustments for Gestational Age and Birth Weight

eTable 5. Distribution of Triplet and Quadruplet Births in the Assisted Reproductive Technology (ART) and Control Groups

eFigure 1. A Comprehensive Overview of the Complex Causal Relationships Between Assisted Reproductive Technology (ART) Exposure and the Development of Atopic Diseases

eFigure 2. Hazard Ratio of Developing Atopic Diseases in Relation to Intracytoplasmic Sperm Injection (ICSI), Adjusted for Cofactors by Cox Proportional Hazards Regression Models (Analysis Limited to ART-Conceived Children)

eFigure 3. Hazard Ratio of Developing Atopic Diseases in Relation to Fresh or Frozen Embryo, Adjusted for Cofactors by Cox Proportional Hazards Regression Models (Analysis Limited to ART-Conceived Children)

This supplemental material has been provided by the authors to give readers additional information about their work.

© 2025 Hsieh YC et al. *JAMA Network Open*.

eTable 1. Specific *ICD-9-CM* and *ICD-10-CM* Diagnostic Codes Employed in This Analysis

Disease	ICD-9-CM	ICD-10-CM
Asthma	493	J45
Allergic rhinitis	477	J30
Atopic dermatitis	691	L20
Gestational hypertension	642.3	O13, O16
Pre-eclampsia or eclampsia	624.4, 624.5, 624.6	O11, O14, O15
Gestational diabetes mellitus	648.0, 648.8,	O24

© 2025 Hsieh YC et al. *JAMA Network Open*.

eTable 2. Definitions and Measurement of Covariates

Covariate	Definition and Measurement
**Maternal age**	Maternal age was measured at the time of the child's birth.
**Family income**	Family income was measured based on the insurance premium category assigned to the family at the time of the child's birth.
**Urbanization**	Urbanization was measured using data from the National Health Insurance Research Database (NHIRD), based on the child's registered place of residence at the time of birth.
**History of paternal diseases**	Paternal history of atopic diseases was considered present if at least one inpatient admission or three or more outpatient visits with the corresponding ICD codes during the study period.
**History of maternal diseases**	Maternal history of atopic diseases was considered present if at least one inpatient admission or three or more outpatient visits with the corresponding ICD codes during the study period.

© 2025 Hsieh YC et al. *JAMA Network Open*.

eTable 3. Interaction Tests for Subgroup Analyses of ICSI Use, Embryo Type, and Their Interaction With the Risk of Atopic Disease Development Using Cox Proportional Hazards Regression Models**

	ICSI vs No ICSI, *p v*alue	Fresh vs Frozen Embryo, *p v*alue	ICSI × Embryo type Interaction, *p v*alue
Asthma	0.45	0.72	0.60
Allergic rhinitis	0.50	0.65	0.35
Atopic dermatitis	0.78	0.85	0.92

* ICSI: Intracytoplasmic Sperm Injection; **Model adjusted for family income, urbanization, history of paternal atopic disease, history of maternal atopic disease, pregnancy-related complication, mode of delivery, neonatal gender, birth weight and gestational age

© 2025 Hsieh YC et al. *JAMA Network Open*.

eTable 4. Sensitivity Analyses of the Association Between Artificial Reproductive Technology and Atopic Diseases Stratified by Singleton and Multiple Births, with Alternative Adjustments for Gestational Age and Birth Weight*

Adjusted for Gestation Age

Atopic Disease	Singleton+ Multiple								Singleton			Multiple		
	ART group, n (%)	Control group, n (%)	cHR	95%CI	*p*	aHR	95%CI	*p*	aHR	95%CI	*p*	aHR	95%CI	*p*
**Asthma**	4732 (33.9)	16183 (29.0)	1.22	1.18- 1.26	<0.001	1.14	1.10- 1.18	<0.001	1.15	1.11- 1.20	<0.001	1.00	0.89- 1.12	0.995
**Allergic rhinitis**	8740 (62.6)	31916 (57.2)	1.17	1.15- 1.20	<0.001	1.15	1.12- 1.18	<0.001	1.16	1.13- 1.19	<0.001	1.05	0.97- 1.15	0.238
**Atopic dermatitis**	4519 (32.4)	16991 (30.4)	1.07	1.04- 1.11	<0.001	1.08	1.05- 1.12	<0.001	1.08	1.04- 1.12	<0.001	1.12	0.99- 1.27	0.082

Adjusted for Birth weight

Atopic Disease	Singleton+ Multiple								Singleton			Multiple		
	ART group, n (%)	Control group, n (%)	cHR	95%CI	*p*	aHR	95%CI	*p*	aHR	95%CI	*p*	aHR	95%CI	*p*
**Asthma**	4732 (33.9)	16183 (29)	1.22	1.18- 1.26	<0.001	1.14	1.10- 1.18	<0.001	1.15	1.11- 1.20	<0.001	1.01	0.90- 1.13	0.930
**Allergic rhinitis**	8740 (62.6)	31916 (57.2)	1.17	1.15- 1.20	<0.001	1.15	1.12- 1.18	<0.001	1.16	1.13- 1.19	<0.001	1.06	0.97- 1.15	0.201
**Atopic dermatitis**	4519 (32.4)	16991 (30.4)	1.07	1.04- 1.11	<0.001	1.08	1.05- 1.12	<0.001	1.08	1.04- 1.12	<0.001	1.12	0.99- 1.27	0.085

Adjusted for both Gestation Age and Birth weight

Atopic Disease	Singleton+ Multiple								Singleton			Multiple		
	ART group, n (%)	Control group, n (%)	cHR	95%CI	*P-value*	aHR	95%CI	*p*	aHR	95%CI	*p*	aHR	95%CI	*p*
**Asthma**	4732 (33.9)	16183 (29)	1.22	1.18- 1.26	<0.001	1.13	1.09- 1.18	<0.001	1.15	1.11- 1.19	<0.001	1.00	0.89- 1.12	1.000
**Allergic rhinitis**	8740 (62.6)	31916 (57.2)	1.17	1.15- 1.20	<0.001	1.15	1.12- 1.18	<0.001	1.16	1.13- 1.19	<0.001	1.06	0.97- 1.15	0.224
**Atopic dermatitis**	4519 (32.4)	16991 (30.4)	1.07	1.04- 1.11	<0.001	1.08	1.05- 1.12	<0.001	1.08	1.04- 1.12	<0.001	1.12	0.99 1.27	0.079

*All models were additionally adjusted for family income, level of urbanization, parental history of atopic diseases (maternal and paternal), pregnancy-related complications, mode of delivery, and neonatal sex.

© 2025 Hsieh YC et al. *JAMA Network Open*.

eTable 5. Distribution of Triplet and Quadruplet Births in the Assisted Reproductive Technology (ART) and Control Groups

	Triplets	Quadruplets
ART group	90	0
Control Group	19	1

© 2025 Hsieh YC et al. *JAMA Network Open*.

eFigure 1. A Comprehensive Overview of the Complex Causal Relationships Between Assisted Reproductive Technology (ART) Exposure and the Development of Atopic Diseases*

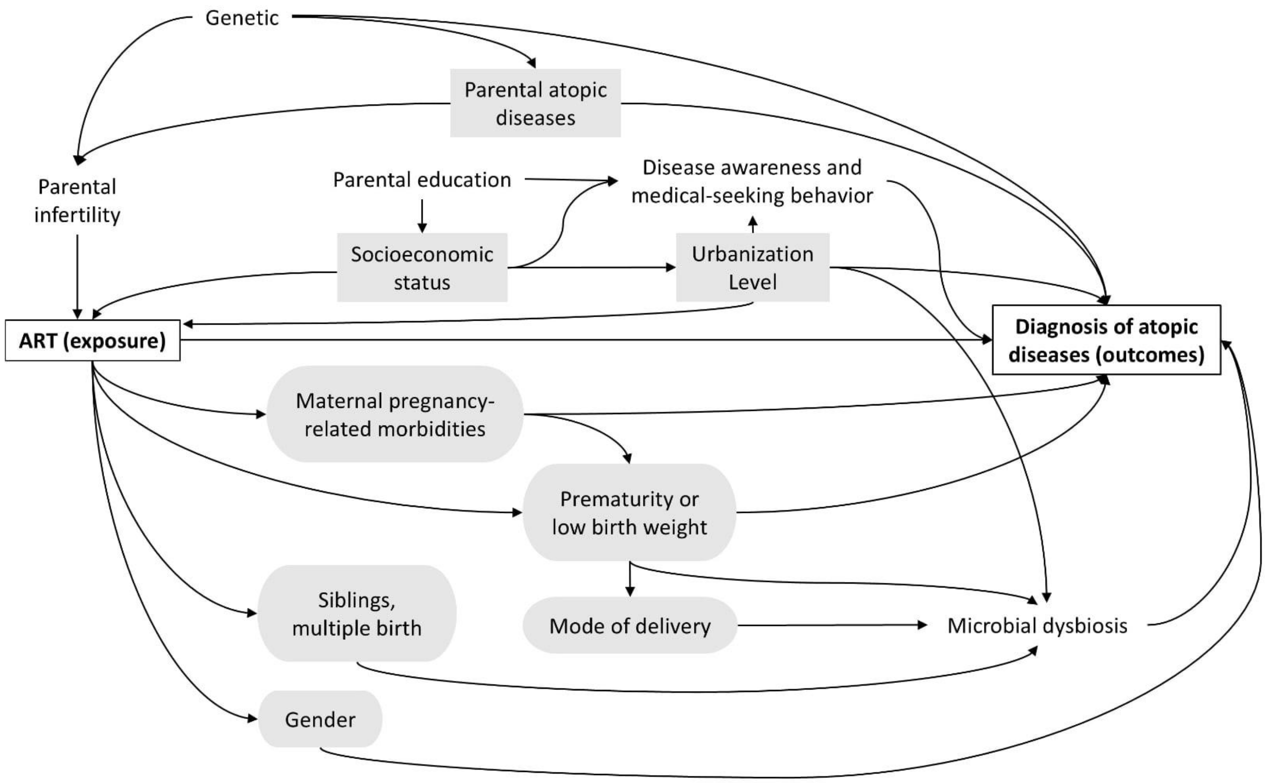

* The grey-shaded rectangular nodes represent potential confounders we choose in the regression model, while the grey-shaded elliptical nodes indicate mediators along the causal pathway from exposure to outcome.

© 2025 Hsieh YC et al. *JAMA Network Open*.

eFigure 2. Hazard Ratio of Developing Atopic Diseases in Relation to Intracytoplasmic Sperm Injection (ICSI), Adjusted for Cofactors by Cox Proportional Hazards Regression Models* (Analysis Limited to ART-Conceived Children)

	Use ICSI N(%)	No use ICSI N(%)	Adjusted Hazard Ratio(95% CI)	*P* Value
**Asthma**	2487 (33.3)	2245(34.6)	1.04(0.98-1.10)	0.18
**Allergic rhinitis**	4663 (62.5)	4077(62.8)	0.99(0.95-1.03)	0.68
**Atopic dermatitis**	2369 (31.8)	2150(33.1)	1.04(0.98-1.11)	0.17

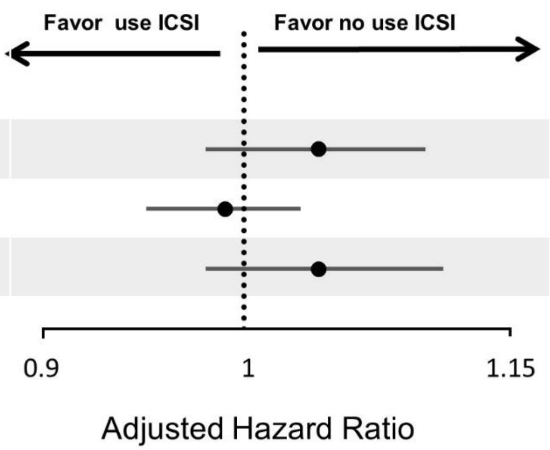

*CI: confidence interval; Model adjusted for family income, urbanization, history of paternal atopic disease, history of maternal atopic disease, pregnancy-related complication, mode of delivery, neonatal gender, multiple births, birth weight and gestational age

© 2025 Hsieh YC et al. *JAMA Network Open*.

eFigure 3. Hazard Ratio of Developing Atopic Diseases in Relation to Fresh or Frozen Embryo, Adjusted for Cofactors by Cox Proportional Hazards Regression Models* (Analysis Limited to ART-Conceived Children)

	Frozen Embryo N(%)	Fresh Embryo N(%)	Adjusted Hazard Ratio(95% CI)	*P* Value
**Asthma**	694 (33.9)	4038(33.9)	0.96(0.89-1.05)	0.37
**Allergic rhinitis**	1177 (57.4)	7563(63.5)	1.12(1.06-1.19)	<0.001
**Atopic dermatitis**	659 (32.1)	3860(32.4)	1.01(0.93-1.10)	0.79

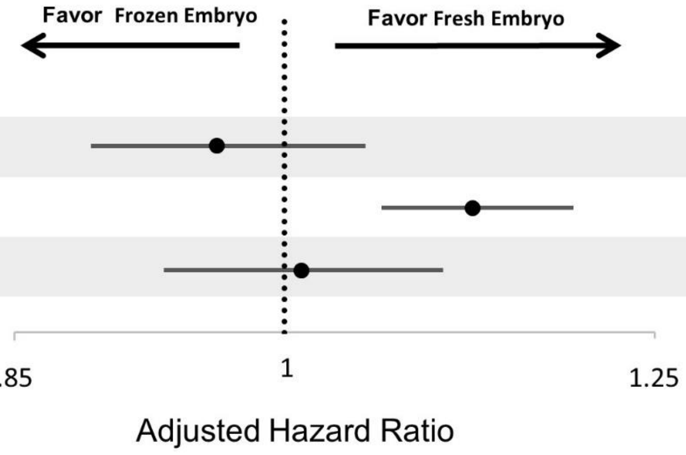

*CI: confidence interval; Model adjusted for family income, urbanization, history of paternal atopic disease, history of maternal atopic disease, pregnancy-related complication, mode of delivery, neonatal gender, multiple births, birth weight and gestational age

© 2025 Hsieh YC et al. *JAMA Network Open*.
