# Supplementary material for: Atopic Disease Development in Offspring Conceived via Assisted Reproductive Technology
Source: JAMA Netw Open. 2025 Dec 30;8(12):e2551690. doi: 10.1001/jamanetworkopen.2025.51690 (PMC12754677; doi:10.1001/jamanetworkopen.2025.51690)
Supplement: Supplement 2. — Data Sharing Statement [file jamanetwopen-e2551690-s002.pdf]

Data Sharing Statement

Hsieh. Atopic Disease Development in Offspring Conceived via Assisted Reproductive Technology. *JAMA Netw Open*. Published December 30, 2025. doi:10.1001/jamanetworkopen.2025.51690

Data
Data available: No

Additional Information
Explanation for why data not available: To protect patient confidentiality and ensure the reliability of the databases, investigators are required to conduct onsite analyses at the Health and Welfare Data Science Center in Taiwan via a remote connection to the Ministry of Health and Welfare servers. Dr. Ching-Heng Lin (epid@vghtc.gov.tw) had full access to all the data in the study and takes responsibility for the integrity of the data and the accuracy of the data analysis.
